# Individuals at risk for Alzheimer’s disease show differential patterns of ERP brain activation during odor identification

**DOI:** 10.1186/1744-9081-8-37

**Published:** 2012-07-31

**Authors:** Charlie D Morgan, Claire Murphy

**Affiliations:** 1 Department of Psychology, San Diego State University, San Diego, CA, 92120, USA; 2 University of California San Diego Medical Center, San Diego, CA, 92120, USA; 3 SDSU/UCSD Joint Doctoral Program, 6363 Alvarado Ct., Suite 101, San Diego, CA, 92120-4913, USA

**Keywords:** Alzheimer’s disease, Apolipoprotein E, Olfactory event-related potentials, Age, Smell impairment, Olfaction

## Abstract

**Background:**

Studies suggest that older adults at risk of developing Alzheimer’s disease may show olfactory processing deficits before other signs of dementia appear.

**Methods:**

We studied 60 healthy non-demented individuals, half of whom were positive for the genetic risk factor the Apolipoprotein E ɛ4 allele, in three different age groups. Event-related potentials to visual and olfactory identification tasks were recorded and analyzed for latency and amplitude differences, and plotted via topographical maps.

**Results:**

Varying patterns of brain activation were observed over the post-stimulus epoch for ɛ4- versus ɛ4+ individuals on topographical maps. Individuals with the ɛ4 allele demonstrated different ERP peak latencies during identification of olfactory but not visual stimuli. High correct ApoE classification rates were obtained utilizing the olfactory ERP.

**Conclusions:**

Olfactory ERPs demonstrate functional decline in individuals at risk for Alzheimer’s disease at much earlier ages than previously observed, suggesting the potential for pre-clinical detection of AD at very early stages.

## 

Alzheimer’s disease is a neurologic disorder accompanied by progressive memory loss, cognition loss and functional decline [1]. The cause or causes of AD are not yet known and definitive diagnosis can only be made via postmortem autopsy or, while living, a brain biopsy. The greatest risk factor for development of AD is advancing age. Genetic research has confirmed that the ɛ4 allele of the apolipoprotein E (ApoE) gene is the strongest genetic risk factor for AD [2-5]. Inheritance of a single ApoE ɛ4 variant increases a persons risk of developing AD by a factor of three in men and four in women, and having two copies of the ɛ4 allele increases risk up to 15-fold compared to persons without the ɛ4 variant [6,7]. Presence of the ɛ4 allele increases the risk but does not guarantee future development of AD [8].

Studies have found olfactory dysfunction in AD including impairment in olfactory threshold sensitivity, odor identification, odor recognition memory, remote memory for odors, and odor fluency for review see [9]. Regions of the brain involved in the processing of olfactory information, such as the entorhinal cortex, prepiriform cortex, and the anterior olfactory nucleus show increased neuritic plaques and neurofibrillary tangles in AD, as well as cell loss, granulovacuolar degeneration and tangles in the olfactory bulb [4,10-15]. The neuropathological changes associated with AD have been shown to affect the primary regions of the brain involved in olfaction but have less effect on other primary sensory areas [16]. Greater hippocampal atrophy has been reported in non-demented ɛ4+ individuals compared to ɛ4- controls [17]. Studies of persons with the ɛ4 allele have also demonstrated olfactory deficits in odor identification [18], odor detection [19] and odor memory [20], as well as odor recognition memory [21]. Odor identification appears to be particularly sensitive to cognitive changes associated with dementia. Correct classification rates of 100% have been obtained between persons at risk for AD from controls utilizing an odor identification test [22]. ɛ4+ individuals demonstrate significantly poorer odor identification than ɛ4- nondemented older adults [18,23]. Odor identification abilities declined more rapidly in ɛ4+ persons than ɛ4- persons over a four year time period while during the same time period there was no significant change in odor threshold, picture identification, or DRS scores [24]. Odor identification has been shown to be directly related to left hippocampal volume and to AD pathology in the brain [25,26]. Given that areas of the brain that process olfactory information are some of the earliest affected in AD and those at risk for AD, olfactory changes may be some of the earliest signs of the disease in the preclinical phase.

Neuroimaging studies have suggested a functional recruitment hypothesis of age-related compensatory changes where those with AD and those at risk for AD utilize additional cognitive resources to bring memory-related performance to normal levels [27-33]. Persons with a positive family history (FH) of AD and those with both FH and the ɛ4 allele had greater activation predominantly in the bilateral posterior cingulate/precuneus, bilateral temporoparietal junction, and bilateral prefrontal cortex [34]. ApoE+ individuals produced greater brain activation in the bilateral fusiform gyri, right superior parietal cortex, left pyramis/uvula, left middle frontal gyrus, and medial frontal gyrus [29]. Similarly, participants diagnosed with Mild Cognitive Impairment or AD demonstrate greater activation in the frontal areas of the brain [35]. These studies suggest the potential for detection of AD and early preclinical stages using measures of brain response.

Brain activity can be measured from the surface of the scalp via the electroencephalogram and more specifically the event-related potential (ERP), a measure that is exquisitely sensitive to the timing of the brain’s response. Olfactory event-related potentials (OERPs) recorded in relation to olfactory stimulation has demonstrated sensitivity to subtle changes in olfactory functioning associated with aging, disease, and ApoE status [36-45]. OERPs require odor stimulation via specially built olfactometers which control the exact timing of stimulus onset while avoiding simultaneous stimulation of other sensory modalities such as stimulation of the trigeminal system [39,46-49]. These olfactometers also warm and humidify the air stream in order to prevent somatosensory cues. Reaction times to odors vary based on the stimulus and subject characteristics but range from 800-900 ms [50]. Neuronal recovery time of the olfactory system is much longer than other sensory systems [38,51,52]. Auditory and visual stimuli can be presented every 2–3 seconds in ERP research without significant adaptation [53-55] while in the olfactory system inter-stimulus intervals of 30–45 seconds are required. This slower neuronal recovery is partially due to olfactory receptor cells that rapidly adapt and slowly recover [56] and partially due to habituation [52]. Given longer inter-stimulus intervals in olfactory stimulation, fewer trials are presented than in other systems in order to reduce potential subject fatigue and loss of vigilance. A narrower filter is also applied when processing the ERP data to compensate for the smaller number of trials.

The early components of the OERP, the N1, P2, and N2 are considered exogenous sensory components that have been associated with odor threshold and odor identification [38,49,57]. The P3 component in general represents endogenous processing of a stimulus, reflecting both stimulus classification speed and the ability to attend to and evaluate a stimulus [58,59]. OERP P3 latency correlates with neuropsychological tests that measure memory and cognitive processing speed [60]. Several studies have demonstrated increased OERP peak latencies associated with aging [36,38,39,41,60]. Older males produced significantly smaller OERP peak amplitudes than older females when utilizing relatively short inter-stimulus intervals, suggesting greater olfactory impairments in males [38]. Studies of the OERP have further documented olfactory deficits in AD [61], specifically longer P2 and P3 latencies in AD patients compared to controls. These latency measures also correlate significantly with dementia status as measured by the Dementia Rating Scale (DRS). Importantly, studies utilizing the OERP with persons at risk for AD, due to the ApoE ɛ4 allele, have also demonstrated differences. ɛ4+ non-demented older adults produced significantly longer OERP latencies than age-matched ɛ4- individuals [62]. Additionally, high sensitivity and specificity was obtained in classifying ɛ4+ and ɛ4- individuals based on OERP latency alone. Utilizing a cross-modal odor recognition memory task differential brain activity was observed between ApoE groups, such that ɛ4- participants differed from ɛ4+ participants in activation of the frontal electrode sites, supporting the compensatory hypothesis [63].

This study examines OERPs in an odor identification task compared to a picture identification task in three separate age groups and in persons positive and negative for the ɛ4 allele. We hypothesized that ɛ4+ individuals would demonstrate differing topographical patterns of brain activation compared to ɛ4- individuals as measured by the ERP. As in previous studies we also hypothesized that ɛ4+ older adults would produce longer OERP peak latencies than ɛ4- participants and that this difference would be greater than differences measured with an odor threshold test and a traditional odor identification test (San Diego Odor Identification Test).

## Methods and materials

### Participants

Participants were 60 adults divided into three age groups, Young Adults (10 M, 10F, Mean age = 22.8 years), Middle Age Adults (10 M, 10F, Mean Age = 50.5), and Older Adults (10 M, 10F, Mean age = 70.7). Half of each group were positive for the ɛ4 allele. Table 1 presents demographic variables by age and ApoE groups. Participants were recruited from the general community, from San Diego State University, and from an ongoing subject pool at the Lifespan Human Senses Laboratory. The research was approved by IRBs at San Diego State University and the University of California, San Diego and subjects gave informed consent. All participants were screened for odor sensitivity via odor threshold test and odor identification test and any participants with threshold scores lower than 4, or odor identification scores less than 3, were excluded from the study [22,64,65]. Participants were screened for cognitive impairment using the Dementia Rating Scale, and any participants scoring less than 133 were excluded from the study [66]. Genetic DNA was obtained from each subject using buccal swab of cheek cells and analyzed for the APOE genotype at an offsite laboratory as described in [67]. Data from 40 of these participants have previously been published [68].

**Table 1:** Means and Standard Deviations for demographic, behavioral and performance data for each age and ApoE group

	**ApoE Negative**			**ApoE Positive**		
	**Young**	**Middle**	**Older**	**Young**	**Middle**	**Older**
Age (years)	22.6 (2.0)	50.7 (1.7)	71.2 (3.6)	23.1 (2.3)	50.2 (4.5)	70.2 (2.9)
Education (years)	14.7 (2.8)	14.9 (2.5)	16.1 (2.3)	14.9 (1.7)	15.2 (2.3)	14.8 (3.2)
Dementia Rating Scale Score (144 max)	142.4 (2.0)	139.8 (4.8)	140.3 (2.9)	140.3 (5.0)	140.8 (4.1)	141.5 (2.1)
Odor Threshold (dilution steps, 9 max)	7.4 (1.3)	6.7 (1.1)	6.3 (1.4)	7.3 (1.5)	6.8 (1.9)	4.4 (1.3)
Odor Identification Test (6 max)	5.9 (0.3)	4.9 (1.2)	5.0 (1.3)	5.6 (0.5)	4.8 (1.4)	4.3 (1.2)
OERP # Correctly Identified (28 max)	20.1 (4.3)	19.9 (6.1)	18.7 (4.2)	18.3 (3.9)	15.5 (3.7)	17.6 (4.0)
VERP # Correctly Identified (28 max)	21.2 (7.8)	26.0 (2.1)	25.7 (2.8)	22.7 (4.0)	22.1 (6.5)	26.3 (2.2)

## Procedure

### San Diego Odor Identification Test (SDOIT)

The San Diego Odor Identification Test [22,57,69] consists of 8 common household odors (e.g. chocolate, coffee) presented in opaque jars. A set of 20 line drawings of the 8 odors and 14 distractors, presented in an array, were placed in front of each participant. Participants smelled the odors birhinically in random order and chose the odor from the array of drawings. Verbalizing the name of the odor or pointing to the picture of the odor were both acceptable ways of responding. Total number correct of the 6 most commonly identified odors was used for analysis.

### ERP stimulus presentation

Olfactory stimulation was performed via computer controlled olfactometer incorporating designs of previous olfactometers [39,46-49]. Odors were presented utilizing a single stimulus paradigm for 200 msec and an interstimulus interval (ISI) of 30 sec. Participants employed Velopharyngeal closure to restrict breathing to the mouth and thereby maintain a constant odorant flow rate [46,48,70]. Fourteen separate odors (banana, rose, cinnamon, peanut butter, baby powder, mustard, chocolate, pine, lemon, orange, vanilla, coffee, leather, wintergreen) were presented twice each in pseudo-randomized order. Six of these odors were chosen in order to replicate the most identifiable odors from the San Diego Odor Identification Test [22,57,69]. All odorants were undiluted and two drops of each odorant were placed into the olfactometer before each subject session. After each odor presentation the participants were asked to identify the odor via button press from a list of four written options presented on the computer screen in front of them and their responses were recorded electronically.

In a separate experimental session on the same testing day, 28 visual line drawings of objects from the Boston Naming Test [71] were presented on a computer screen in front of the subject. Duration of each stimulus was 200 msec with 30 sec ISIs. As in the olfactory identification task the subjects were asked to identify each picture via button press from a list of four written options on the computer screen. Both olfactory and visual stimuli were presented via Compumedics STIM^2^ software. Order of experimental presentation, olfactory or visual, was randomized across subjects so that some subjects received the visual experiment first, and some the olfactory experiment first.

### ERP recording

Olfactory and visual ERPs were obtained via Compumedics™ 64-electode AG/AG/CL sintered Quick-Cap and Quick-Cell system, amplified via Synamps 2 amplifiers, and recorded on computer hard disk via the Neuroscan software package. Electrode impedances were kept below 10 kΩ. At the time of recording the EEG data were digitized at 500 Hz through a 0.1 to 30 Hz bandpass filter. Offline, the data were further filtered through a 0.1 to 6 Hz bandpass filter. Artifactual eyeblink activity was recorded and corrected offline via the Neuroscan software utilizing the Occular Artifact Reduction method within the software. ERP trials that included other types of artifactual activity were excluded using both automated exclusion (e.g. excluding all trials with voltage ranges larger than 50 μV) and by visually inspecting each trial prior to averaging . Ongoing EEG activity was recorded throughout the experiment and then trials epoched offline to 500 msec pre-stimulus and 1500 msec post-stimulus. Baseline corrected trials were then averaged. Peak amplitudes were measured from the pre-stimulus baseline to maximum peak amplitude. Latency windows from previous OERP studies [38,39] were used as guidelines to identify peak components. Peaks were picked blindly as to age, gender, and APOE status in order to avoid experimenter bias.

## Results

### Demographics and screening measures

Table 1 summarizes demographic and screening measures. Within each age group there were no significant differences in mean participant age between ApoE+ and ApoE- participants (p >.05). Dementia Rating Scale scores did not differ significantly between ApoE groups or age groups (p >.05) and all participants’ DRS scores were in the range of normal cognitive functioning. Analysis of odor threshold test performance revealed that all participants scored in the normal range of olfactory functioning. While all participants were normosmic, the older participant group exhibited poorer olfactory threshold scores than the middle and young age groups (F(2,57) = 8.93, p <.001, η^2^ = .24), with no interaction of ApoE status.

### Odor and picture identification

Table 1 shows identification scores by age and ApoE groups. Analysis of the San Diego Odor Identification Test revealed no significant main effects or interaction effects involving ApoE status (p >.05). It did demonstrate a significant main effect of age (F(2,57) = 6.04, p <.01, η^2^ = .18) with young participants correctly identifying more odors than both the middle and older age groups (p <.05).

Analysis of correctly identified number of odors from the Odor Identification ERP task revealed a main effect of ApoE status collapsed across age groups (F(1,54) = 4.54, p <.05) η^2^ = .08), such that ɛ4- participants correctly identified more odors than ɛ4+ participants. The number of correctly identified odors did not differ significantly by ApoE status when each age group was analyzed separately, (suggesting that this effect is small when the screening measures are applied). Analysis of correctly identified pictures in the Picture Identification ERP task revealed a significant main effect of age group (F(2,54) = 3.60, p <.05, η^2^ = .12), such that older participants correctly identified more pictures than young participants (p <.05). The effect of ApoE status was not significant for Picture Identification.

### Topographical displays of ERP activity

**Figure 1:**
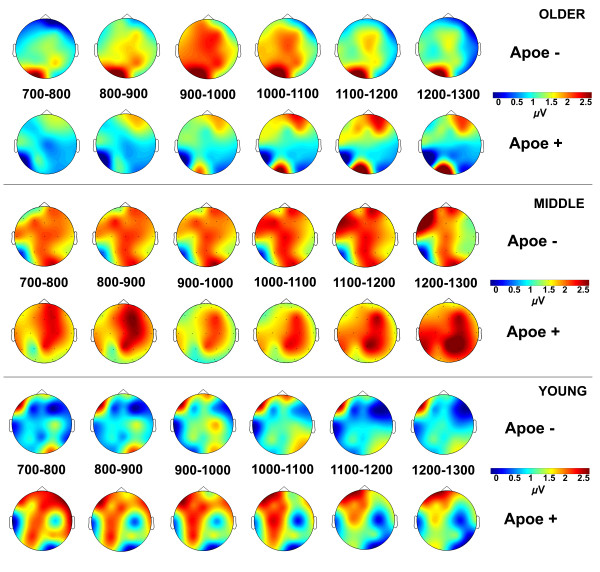
Topographical representation of OERP amplitudes in μV across 19 electrode sites by age and ApoE groups for 700-1300 ms post-stimulus time intervals.

Figure 1 illustrates topographical distributions of OERP amplitudes in μV over the post-stimulus time interval from 700 ms through 1300 ms by age and ApoE groups. For each of 19 electrodes (FP1, FP2, F7, F3, F2, F4, F8, T7, C3, CZ, C4, T8, P7, P3, P2, P4, P8, O1, O2) amplitudes were averaged over the 100 ms time intervals (e.g. 700-800 ms) and input into graphing software in order to visually display brain activity across the scalp for each age and APOE group. Given no significant ApoE effects in the visual modality only olfactory ERP topographies are shown. In the young group the OERP topographical maps show greater brain activation in the ɛ4+ participants compared to ɛ4- participants, over the left hemisphere electrodes, and particularly over parietal electrodes, that decreases after 1100 ms. Middle age ɛ4+ and ɛ4- individuals demonstrated similar topographies, however ɛ4+ participants showed somewhat more activity over the right hemisphere electrodes compared to left, in the 900-1100 ms range, whereas ɛ4- participants showed more central electrode activity across the recording epoch. The greatest differences can be observed in the older groups where ɛ4- participants showed an increase in activation over left and central electrode sites between 900-1100 ms, and ɛ4+ participants showed relatively less overall activation during that time period, but increasing activation over right frontal electrodes between 1000-1200 ms. Overall the topographical maps clearly illustrate that brain activity related to olfactory processing differs not only by age, but more importantly by ApoE status, and the activity differentially changes over the post-stimulus time period.

### Event Related Potentials (ERPs)

Repeated measures multivariate analyses of variance (MANOVAs) were performed for each ERP component (N1, P2, N2, P3) and olfactory and visual modalities were analyzed separately for each peak amplitude and latency. Greenhouse-Geisser corrections were applied to all MANOVAs. Significant main effects and interactions were further analyzed with post hoc Newman Keuls Multiple Range Tests (alpha 0.05). Significant visual and olfactory ERP effects are summarized in Table 2.

**Table 2:** Summary of analyses performed and effect sizes for peak component amplitudes and latencies

**Peak measures**	**Amplitude**				**Latency**			
	**N1**	**P2**	**N2**	**P3**	**N1**	**P2**	**N2**	**P3**
*Picture ID ERPs*								
Age (A)	-	-	-	-	-	-	-	*(η=.15)
ApoE Status (S)	-	-	-	-	-	-	-	-
Electrode (E)	-	-	-	*(η=.07)	-	-	*(η=.06)	*(η=.14)
A x S	-	-	-	-	-	-	-	-
A x E	-	-	-	-	-	-	-	-
S x E	-	-	-	-	-	-	-	-
A x S x E	-	-	-	-	-	-	-	-
*Odor ID ERPs*								
Age (A)	-	-	*(η=.12)	-	***(η=.70)	***(η=.68)	***(η=.64)	***(η=.76)
ApoE Status (S)	-	-	-	-	***(η=.27)	***(η=.22)	-	-
Electrode (E)	-	-	-	-	-	-	-	-
A x S	-	-	-	-	*(η=.15)	***(η=.26)	**(η=.23)	***(η=.34)
A x E	-	-	-	-	-	-	-	-
S x E	-	-	-	-	-	-	-	-
A x S x E	-	-	-	-	-	-	-	-

* p <0.05.

**p <0.01.

***p <0.001.

### Picture identification ERPs

Visual N1, P2, and N2 amplitudes demonstrated no significant main or interaction effects (p >.05). Visual P3 amplitude demonstrated a significant main effect for electrode site (F(2,108) = 4.28, p <.05, η^2^ = .07) such that the Cz and Pz electrode sites produced larger P3 amplitudes than the Fz site.

**Figure 2:**
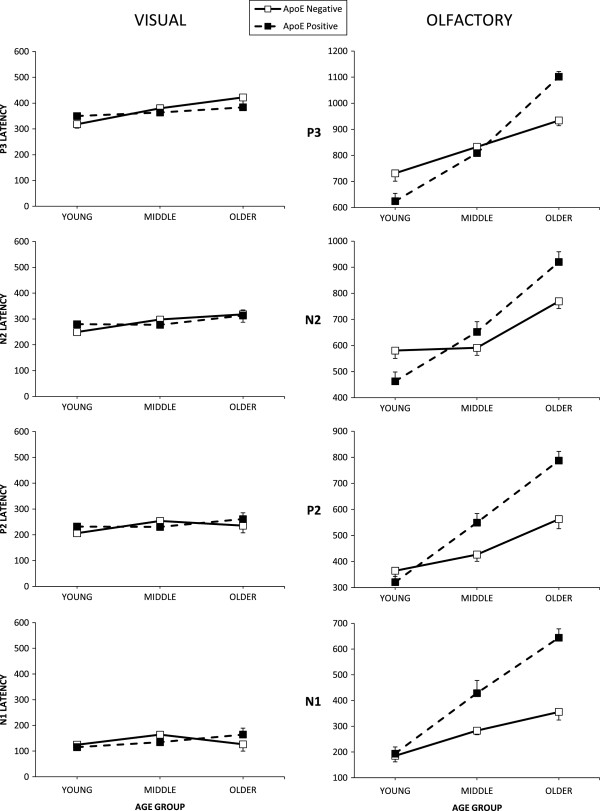
**Visual and olfactory average N1, P2, N2, and P3 latencies by age and ApoE groups.** Error bars represent the standard error of the mean (SEM).

Figure 2 illustrates visual ERP peak latencies. Visual N1 and P2 latencies did not demonstrate significant main or interaction effects (p >.05). Visual N2 latency demonstrated a significant main effect of electrode site (F(2,108) = 3.50, p <.05, η^2^ = .06) with the Pz electrode site producing shorter N2 latencies than the Fz electrode site. Visual P3 latency also revealed a significant main effect of electrode site (F(2,108) = 8.78, p <.01, η^2^ = .14) with the Cz and Pz electrode sites recording significantly longer latencies than the Fz site (p <.01). Visual P3 latency also showed a significant main effect of age group (F(2,54) = 4.94, p <.05, η^2^ = .15) with older adults producing significantly longer visual P3 latencies than young adults (p <.01).

### Odor identification ERPs

Olfactory N1, P2, and P3 amplitudes demonstrated no significant main or interaction effects (p >.05). Olfactory N2 amplitude demonstrated a significant main effect of age (F(2,54) = 3.62, p <.05, η^2^ = .12) with older age participants producing significantly more negative N2 amplitudes than middle age participants.

Figure 2 illustrates olfactory ERP peak latencies. Olfactory N1, P2, N2, and P3 latencies demonstrated significant interaction effects of Age x ApoE status (N1: F(2,54) = 4.73, p <.05, η^2^ = .15; P2: F(2,54) = 9.34, p <.001, η^2^ = .26; N2: F(2,54) = 8.18, p <.01, η^2^ = .23; P3: F(2,54) = 14.11, p <.001, η^2^ = .34). Post hoc analyses of the interaction effects revealed that in the young group those negative for the ɛ4 allele produced significantly longer N2 (η^2^ = .26), and P3 (η^2^ = .29), latencies. In the middle age group those positive for the ɛ4 allele produced significantly longer N1 (η^2^ = .31), and P2 (η^2^ = .31) latencies. In the older group those positive for the ɛ4 allele produced significantly longer latencies for all components N1 (η^2^ = .50), P2 (η^2^ = .52), N2(η^2^ = .35), and P3(η^2^ = .59). For N1 and P3 latencies, in both ApoE groups, older participants produced significantly longer latencies than middle age participants and middle age participants produced longer latencies than young participants. For P2 and N2 latencies in ɛ4+ participants, this same pattern was observed, however in ɛ4- participants young adults did not differ significantly from middle age participants, but both young and middle age participants produced shorter latencies than older participants.

Correlational analyses were performed between the ERP odor ID performance and average (Fz, Cz, Pz) peak latencies (N1, P2, N2, P3). When all ages and ApoE groups were combined together odor ID performance marginally correlated with N1 latency (r = −.28, p <.05). Correlational analyses were also performed for each age x ApoE group separately. The only significant correlation between odor ID performance and latency was for N1 latency in the older Apoe+ group (r = −.77, p <.01).

### Logistic regression analyses of OERP variables

In order to better understand the predictive value of the ERP in differentiating ɛ4- and ɛ4+ participants, stepwise logistic regression analysis was performed on olfactory N1, P2, N2, P3 amplitude and latency averaged over Fz, Cz, and Pz. Visual ERP variables were not included in logistic regression analysis because no significant ApoE effects were present. A logistic regression is a type of regression analysis used to predict the outcome of a binary dependent variable (e.g. Apoe+ vs Apoe-) based on one or more predictor variables (e.g. ERP amplitude and latency). The logistic regression analysis outputs predictive classification results including overall correct classification (total percentage of correctly classified individuals), as well as sensitivity (e.g. APOE+ correctly classified as APOE+) and specificity (e.g. APOE- correctly classified as APOE-). In the olfactory modality analysis of all age groups combined revealed that olfactory N1 latency was the most significant single predictor in discriminating between ɛ4+ participants and ɛ4- participants (χ^2^ = 6.07, p <.05) resulting in an overall correct classification rate of 70.0% (Sensitivity = 66.7%, Specificity = 73.3%). When P3 latency was also added to N1 amplitude in the model (χ^2^ = 8.06, p <.01) overall correct classification rate increased to 76.7% (Sensitivity = 80%, Specificity = 73.3%). Logistic regressions were also performed for each age group separately in order to better understand the effects of ApoE status within each age group. In the young participant group N2 amplitude was the most significant predictor (χ^2^ = 7.79, p <.01) resulting in an overall classification rate of 65.0% (Sensitivity = 70.0%, Specificity = 60.0%). In the middle age group P2 latency was the most significant single predictor (χ^2^ = 6.86, p <.01) resulting in an overall classification rate of 80.0% (Sensitivity = 80.0%, Specificity = 80.0%). When P3 amplitude and P3 latency were also added to the equation with P2 latency (χ^2^ = 20.09, p <.001), the overall classification rate for middle age participants increased to 90.0% (Sensitivity = 90.0%, Specificity = 90.0%). In the older age group P3 latency was the most significant single predictor (χ^2^ = 16.37, p <.001) resulting in an overall classification rate of 90.0% (Sensitivity = 90.0%, Specificity = 90.0%). When N1 latency was also added to the equation with P3 latency (χ^2^ = 27.73, p <.001), overall classification rate for older participants increased to 100.0% (Sensitivity = 100.0%, Specificity = 100.0%).

## Discussion

This study demonstrated (1) robust odor identification ERP differences based on ApoE status and interactions with age; (2) high correct ApoE classification rates utilizing the OERP that were different for each age group.

A few previous studies have demonstrated ERP impairments in persons with a positive family history of AD, in those diagnosed with early AD, and in individuals with mild cognitive impairment (MCI). MCI is commonly defined as subtle but measurable memory impairment without any other symptoms of dementia. Green et al. [72] demonstrated auditory ERP P3 latency increases in pre-clinical groups of persons with a family history of AD and in those with a family history plus ɛ4+ status. They did not indicate, however, how many of the participants demonstrated this latency difference, or how well this measure correctly classified participants into ɛ4+ or ɛ4- groups. Olichney et al. [73] studied participants diagnosed with MCI utilizing an N400/P600 semantic congruency task and then tracked those participants over time. Participants with abnormal N400 or P600 effects had an 87 to 88% likelihood of progression to dementia within 3 years. They suggest that these N400 abnormalities in MCI may reflect subtle dysfunction of semantic memory processes. Utilizing this method, classification of participants into diagnostic groups was high in sensitivity in MCI who converted to AD (81-94%) less so when applied to all participants (58 to 65%). Chapman et al. [74] used a visual number-letter memory task to study ERPs in participants diagnosed as being in the early stages of AD. Their results suggest that AD deficits may include problems with storage in short-term memory, and that difficulties may lie in the cognitive processing of stimuli that are relevant to the task in which the participant is engaged. Using this method the authors were able to correctly classify 92% of participants into AD or control groups. While these studies are extremely helpful in understanding cognitive changes associated with early stages of dementia, even at the MCI stage, they also raise the possibility of early diagnosis at even earlier, pre-MCI, pre-clinical, stages of AD. A measure that would aid diagnosis of AD many years before any manifestation of other signs or symptoms would be ideal, especially once an effective medication for halting or altering the dementing process is developed.

Illustrated on the ERP topographical maps, the current study demonstrated differing patterns of brain activity recorded over the scalp, depending on ApoE status. fMRI studies on activation have reported mixed results, with some showing increased activation in ɛ4 carriers [30,75], and others demonstrating reduced activation in ɛ4 carriers [76]. The current study demonstrates that patterns of brain activation differ not only by ApoE status, but across age groups, and that the pattern of activation changes across time over the post-stimulus interval of cognitive processing. This suggests that processing of olfactory stimuli is differentially affected by presence or absence of the ɛ4 allele. Additionally these effects change over the lifespan, such that individuals in different decades of life, even young adults, show varying patterns of brain activation.

In the present study, it is important to note that in order to better understand the effects of the ApoE risk factor at points *before* memory problems are present, participants with dementia, early dementia, or mild cognitive impairment, as well as those with anosmia or severe hyposmia, were screened out of the study. Given this rigorous screening process, it is not surprising that no significant ApoE effects were found for the visual picture identification task. Very robust effects of ApoE status were demonstrated for the odor identification ERP task, as well as significant age by ApoE interaction effects. This occurred despite no difference in performance on the Dementia Ratings Scale, odor threshold testing, or the SDOIT by ApoE status. The present study strongly suggests that combining olfactory processing with cognitive processing [odor identification] may be sensitive enough to differentiate very early those with a high likelihood from those with a low likelihood of developing AD, even as early as middle age. This together suggests the ability to identify odors and the speed of odor identification is slowed in the presence of the ɛ4 allele. Picture identification remained intact in ApoE+ participants, both for number of pictures correctly identified and speed with which the pictures were cognitively processed.

Of further note, the present study demonstrated good ApoE classification rates of 80% in the middle age group, with ɛ4+ participants producing significantly longer N1 and P2 latencies than ɛ4- participants. It suggests that ApoE-related olfactory functional decline is taking place at much earlier ages than previously observed, further suggesting that early, pre-clinical, diagnosis of AD may be a real possibility. Consistent with previous OERP studies [62,63], older ɛ4+ participants produced significantly longer OERP peak latencies. In this group 100% classification rates were obtained suggesting a clear delineation in performance between ɛ4+ and ɛ4- participants once they reach ages above 65 years old.

Further research needs to examine the utility of various measures and tasks in the study of pre-clinical AD, in order to capture the pre-dementing processes at the earliest possible stages, and improve diagnostic ability. Olfactory tasks involving cognitive processing, such as the OERP, appear to be very promising in this regard.

## Competing interest

The authors report no biomedical financial interests or potential conflicts of interest.

## Authors’ contributions

CDM and CM designed the study and advised the research assistants through data collection and analysis, and interpreted the data. CDM constructed the olfactometer. CDM and CM wrote and approved the final version of the manuscript.

## References

[B1] American Psychiatric Association Diagnostic and statistical manual of mental disorders, Revised 4th ed 2000 Washington, DC: American Psychiatric Press

[B2] Blacker D The genetics of Alzheimer's disease: progress, possibilities, and pitfalls Harv Rev Psychiatry 1997 5 4 234 237 10.3109/10673229709000306 9427016

[B3] Combarros O Alvarez-Arcaya A Sanchez-Guerra M Infante J Berciano J Candidate gene association studies in sporadic Alzheimer's disease Dement Geriatr Cogn Disord 2002 14 1 41 54 10.1159/000058332 12053131

[B4] Corder EH Saunders AM Strittmatter WJ Schmechel DE Gaskell PC Small GW Roses AD Haines JL Pericakvance MA Gene dose of apolipoprotein E type 4 allele and the risk of Alzheimer's disease in late onset families Science 1993 261 5123 921 923 10.1126/science.8346443 8346443

[B5] Teter B Raber J Nathan B Crutcher KA The presence of apoE4, not the absence of apoE3, contributes to AD pathology J Alzheimers Dis 2002 4 155 163 12226534 10.3233/jad-2002-4305

[B6] Bertram L Tanzi RE The genetic epidemiology of neurodegenerative disease J Clin Invest 2005 115 6 1449 1457 10.1172/JCI24761 15931380 PMC1137006

[B7] Farrer LA Cupples LA Haines JL Hyman B Kukull WA Mayeux R Myers RH Pericakvance MA Risch N vanDuijn CM Effects of age, sex, and ethnicity on the association between apolipoprotein E genotype and Alzheimer disease - A meta-analysis JAMA 1997 278 16 1349 1356 10.1001/jama.1997.03550160069041 9343467

[B8] Burke W Pinsky LE Press NA Categorizing genetic tests to identify their ethical, legal, and social implications Am J Med Genet 2001 106 3 233 240 10.1002/ajmg.10011 11778984

[B9] Murphy C Loss of olfactory function in dementing disease Physiol Behav 1999 66 2 177 182 10.1016/S0031-9384(98)00262-5 10336141

[B10] Averback P 2 new lesions in Alzheimers-disease Lancet 1983 2 8360 1203 6139562 10.1016/s0140-6736(83)91256-4

[B11] Braak H Braak E Frequency of stages of Alzheimer-related lesions in different age categories Neurobiol Aging 1997 18 351 357 10.1016/S0197-4580(97)00056-0 9330961

[B12] Christen-Zaech S Kraftsik R Pillevuit O Kiraly M Martins R Khalili K Miklossy J Early olfactory involvement in Alzheimer's disease Can J Neurol Sci 2003 30 1 20 25 12619779 10.1017/s0317167100002389

[B13] Esiri MM Wilcock GK The Olfactory bulbs in Alzheimers-disease J Neurol Neurosurg Psychiatry 1984 47 1 56 60 10.1136/jnnp.47.1.56 6693914 PMC1027641

[B14] Ohm TG Braak H Olfactory-Bulb Changes in Alzheimers-Disease Acta Neuropathol 1987 73 4 365 369 10.1007/BF00688261 3618129

[B15] Reyes PF Golden GT Fariello RG Fagel L Zalewska M Olfactory pathways in Alzheimer's disease (AD): Neuropathological studies [abstract] Society for Neuroscience 1985 11 168

[B16] Van Hoesen GW Solodkin A Cellular and systems neuroanatomical changes in Alzheimer's disease. In: Calcium hypothesis of aging and dementia. Disterhoft JF, Khachaturian ZS (eds) Proc NY Acad Sci (USA) 1994 747 12 35 10.1111/j.1749-6632.1994.tb44399.x 7847666

[B17] den Heijer T Oudkerk M Launer LJ Van Duijn CM Hofman A Breteler MM Hippocampal, amygdalar, and global brain atrophy in different apolipoprotein E genotypes Neurology 2002 59 746 748 10.1212/WNL.59.5.746 12221169

[B18] Murphy C Bacon AW Bondi MW Salmon DP Apolipoprotein E status is associated with odor identification deficits in nondemented older persons Ann N Y Acad Sci 1998 855 744 750 10.1111/j.1749-6632.1998.tb10654.x 9929680

[B19] Bacon AW Bondi MW Salmon DP Murphy C Very early changes in olfactory functioning due to Alzheimer's disease and the role of apolipoprotein E in olfaction Ann N Y Acad Sci 1998 855 723 731 10.1111/j.1749-6632.1998.tb10651.x 9929677

[B20] Schiffman SS Graham BG Sattely-Miller EA Zervakis J Welsh-Bohmer K Taste, smell and neuropsychological performance of individuals at familial risk for Alzheimer's disease Neurobiol Aging 2002 23 3 397 404 10.1016/S0197-4580(01)00337-2 11959402

[B21] Gilbert PE Murphy C Differences between recognition memory and remote memory for olfactory and visual stimuli in nondemented elderly individuals genetically at risk for Alzheimer's disease Exp Gerontol 2004 39 3 433 441 10.1016/j.exger.2004.01.001 15036403

[B22] Morgan CD Nordin S Murphy C Odor Identification as an Early Marker for Alzheimers-Disease - Impact of Lexical Functioning and Detection Sensitivity J Clin Exp Neuropsychol 1995 17 5 793 803 10.1080/01688639508405168 8557819

[B23] Olofsson JK Nordin S Wiens S Hedner M Nilsson LG Larsson M Odor identification impairment in carriers of ApoE-varepsilon4 is independent of clinical dementia Neurobiol Aging 2010 31 4 567 577 10.1016/j.neurobiolaging.2008.05.019 18619712

[B24] Calhoun-Haney R Murphy C Apolipoprotein epsilon 4 is associated with more rapid decline in odor identification than in odor threshold or Dementia Rating Scale scores Brain Cogn 2005 58 2 178 182 10.1016/j.bandc.2004.10.004 15919549

[B25] Murphy C Jernigan TL Fennema-Notestine C Left hippocampal volume loss in Alzheimer's disease is reflected in performance on odor identification: a structural MRI study J Int Neuropsychol Soc 2003 9 3 459 471 12666770 10.1017/S1355617703930116

[B26] Wilson RS Arnold SE Schneider JA Boyle PA Buchman AS Bennett DA Olfactory Impairment in Presymptomatic Alzheimer's Disease Ann N Y Aca Sci 2009 1170 1 730 735(736) 10.1111/j.1749-6632.2009.04013.x PMC2857767 19686220

[B27] Backman L Andersson JL Lyberg L Winblad B Nordberg A Almkvist O Brain regions associated with episodic retrieval in normal aging and Alzheimer's disease Neurology 1999 52 9 1861 1870 10.1212/WNL.52.9.1861 10371535

[B28] Becker JT Mintun MA Aleva K Wiseman MB Nichols T DeKosky ST Compensatory reallocation of brain resources supporting verbal episodic memory in Alzheimer's disease Neurology 1996 46 3 692 700 10.1212/WNL.46.3.692 8618669

[B29] Bondi MW Houston WS Eyler LT Brown GG fMRI evidence of compensatory mechanisms in older adults at genetic risk for Alzheimer disease Neurology 2005 64 3 501 508 10.1212/01.WNL.0000150885.00929.7E 15699382 PMC1761695

[B30] Bookheimer SY Strojwas MH Cohen MS Saunders AM Pericak-Vance MA Mazziotta JC Small GW Patterns of brain activation in people at risk for Alzheimer's disease N Engl J Med 2000 343 7 450 456 10.1056/NEJM200008173430701 10944562 PMC2831477

[B31] Grady CL McIntosh AR Beig S Keightley ML Burian H Black SE Evidence from functional neuroimaging of a compensatory prefrontal network in Alzheimer's disease J Neurosci 2003 23 3 986 993 12574428 10.1523/JNEUROSCI.23-03-00986.2003 PMC6741917

[B32] Saykin AJ Flashman LA Frutiger SA Johnson SC Mamourian AC Moritz CH O’Jile JR Riordan HJ Santulli RB Smith CA Weaver JB Neuroanatomic substrates of semantic memory impairment in Alzheimer's disease: patterns of functional MRI activation J Int Neuropscyhol Soc 1999 5 5 377 392 10.1017/S135561779955501X PMC4443687 10439584

[B33] Woodard JL Grafton ST Votaw JR Green RC Dobraski ME Hoffman JM Compensatory recruitment of neural resources during overt rehearsal of word lists in Alzheimer's disease Neuropsychology 1998 12 4 491 504 9805319 10.1037//0894-4105.12.4.491

[B34] Seidenberg M Guidotti L Nielson KA Woodard JL Durgerian S Antuono P Zhang Q Rao SM Semantic memory activation in individuals at risk for developing Alzheimer disease Neurology 2009 73 8 612 620 10.1212/WNL.0b013e3181b389ad 19704080 PMC2731619

[B35] Rombouts SA Barkhof F van Meel CS Scheltens P Alterations in brain activation during cholinergic enhancement with rivastigmine in Alzheimer's disease J Neurol Neurosurg Psychiatry 2002 73 6 665 671 10.1136/jnnp.73.6.665 12438467 PMC1757335

[B36] Covington JW Geisler MW Polich J Murphy C Normal aging and odor intensity effects on the olfactory event-related potential Int J Psychophysiol 1999 32 3 205 214 10.1016/S0167-8760(99)00012-4 10437632

[B37] Evans WJ Cui L Starr A Olfactory event-related potentials in normal human subjects: effects of age and gender Electroencephalogr Clin Neurophysiol 1995 95 4 293 301 10.1016/0013-4694(95)00055-4 8529560

[B38] Morgan CD Covington JW Geisler MW Polich J Murphy C Olfactory event-related potentials: older males demonstrate the greatest deficits Electroencephalogr Clin Neurophysiol 1997 104 4 351 358 10.1016/S0168-5597(97)00020-8 9246073

[B39] Morgan CD Geisler MW Covington JW Polich J Murphy C Olfactory P3 in young and older adults Psychophysiology 1999 36 3 281 287 10.1017/S0048577299980265 10352551

[B40] Morgan CD Murphy C Differential effects of active attention and age on event-related potentials to visual and olfactory stimuli Int J Psychophysiol 2010 78 2 190 199 10.1016/j.ijpsycho.2010.07.008 20688110 PMC3086074

[B41] Murphy C Morgan CD Geisler MW Wetter S Covington JW Madowitz MD Nordin S Polich JM Olfactory event-related potentials and aging: normative data Int J Psychophysiol 2000 36 2 133 145 10.1016/S0167-8760(99)00107-5 10742568

[B42] Nordin S Quinonez C Morgan CD Geisler MW Polich J Murphy C Olfactory event-related potentials in young and elderly adults: evaluation of tracking task versus eyes open/closed recording Chem Senses 1999 24 4 459 464 10.1093/chemse/24.4.459 10480682

[B43] Thesen T Wetter S Murphy C Olfactory event-related potential detects age-related changes in olfactory processing with velopharyngeal closure and normal breathing Psychophysiology 2000 37 S97

[B44] Wetter S Murphy C Individuals with Down's syndrome demonstrate abnormal olfactory event-related potentials Clin Neurophysiol 1999 110 9 1563 1569 10.1016/S1388-2457(99)00086-3 10479023

[B45] Wetter S Peavy G Jacobson M Hamilton J Salmon D Murphy C Olfactory and auditory event-related potentials in Huntington's disease Neuropsychology 2005 19 4 428 436 16060817 10.1037/0894-4105.19.4.428

[B46] Kobal G Elektrophysiologische Untersuchungen des menschlichen Geruchsinns 1981 Stuttgart: Thieme

[B47] Lorig TS Elmes DG Zald DH Pardo JV A computer-controlled olfactometer for fMRI and electrophysiological studies of olfaction Behav Res Methods Instrum Comput 1999 31 2 370 375 10.3758/BF03207734 10495824

[B48] Lorig TS The application of electroencephalographic techniques to the study of human olfaction: a review and tutorial Int J Psychophysiol 2000 36 2 91 104 10.1016/S0167-8760(99)00104-X 10742565

[B49] Murphy C Nordin S de Wijk RA Cain WS Polich J Olfactory-evoked potentials: assessment of young and elderly, and comparison to psychophysical threshold Chem Senses 1994 19 1 47 56 10.1093/chemse/19.1.47 8055258

[B50] Overbosch P de Wijk R de Jonge TJ Koster EP Temporal integration and reaction times in human smell Physiol Behav 1989 45 3 615 626 10.1016/0031-9384(89)90082-6 2756054

[B51] Ekman GB Berglund U Berglund B Lindwall T Perceived intensity of odor: a function of time of adaptation Scan J Psychol 1967 8 177 186 10.1111/j.1467-9450.1967.tb01392.x 6079317

[B52] Wilson DA Linster C Neurobiology of simple memory J Neurophysiol 2008 100 2 7 10.1152/jn.90479.2008 18463176

[B53] Polich J P300, probability, and inter-stimulus interval Psychophysiology 1990 27 396 403 10.1111/j.1469-8986.1990.tb02333.x 2236442

[B54] Polich J Probability and inter-stimulus interval effects on the P300 from auditory stimuli Int J Psychophysiol 1990 10 163 170 10.1016/0167-8760(90)90030-H 2272863

[B55] Polich J Niedermeyer E, Lopes da Silva F P300 in clinical applications: meaning, method, and measurement Electroencephalography: Basic Principles, Clinical Applications, and Related Fields 1993 3 Baltimore, MD: Williams and Wilkins 1005 1018

[B56] Moore PA A model of the role of adaptation and disadaptation in olfactory receptor neurons: implications for the coding of temporal and intensity patterns in odor signals Chem Senses 1994 19 1 17 86 10.1093/chemse/19.1.71 8055260

[B57] Murphy C Morgan CD Iqbal K, Sisodia SS, Winblad B Olfactory Function and Event-Related Potentials in Alzheimers Disease Alzheimer’s Disease: Advances in Etiology, Pathogenesis and Therapeutics 2001 Chichester, UK: John Wiley and Sons, Ltd 237 251

[B58] Donchin E Heffley E Hillyard SA Loveless N Maltzman I Ohman A Rosler F Ruchkin D Siddle D Cognition and event-related potentials. II. The orienting reflex and P300 Ann N Y Acad Sci 1984 425 39 57 10.1111/j.1749-6632.1984.tb23522.x 6588858

[B59] Polich J Hoffman LD P300 and handedness: on the possible contribution of corpus callosal size to ERPs Psychophysiology 1993 35 5 497 507 9715094 10.1017/s0048577298970792

[B60] Geisler MW Morgan CD Covington JW Murphy C Neuropsychological performance and cognitive olfactory event-related brain potentials in young and elderly adults J Clin Exp Neuropsychol 1999 21 1 108 126 10.1076/jcen.21.1.108.935 10421006

[B61] Morgan CD Murphy C Olfactory event-related potentials in Alzheimer's disease J Int Neuropsychol Soc 2002 8 6 753 763 10.1017/S1355617702860039 12240739

[B62] Wetter S Murphy C Apolipoprotein E epsilon 4 positive individuals demonstrate delayed olfactory event-related potentials Neurobiol Aging 2001 22 3 439 447 10.1016/S0197-4580(01)00215-9 11378251

[B63] Murphy C Solomon ES Haase L Wang M Morgan CD Olfaction in aging and Alzheimer's disease: event-related potentials to a cross-modal odor-recognition memory task discriminate ApoE epsilon4+ and ApoE epsilon 4- individuals Ann N Y Acad Sci 2009 1170 647 657 10.1111/j.1749-6632.2009.04486.x 19686207 PMC4575288

[B64] Cain WS Gent J Catalanotto FA Goodspeed RB Clinical evaluation of olfaction Am J Otolaryngol 1983 4 4 252 256 10.1016/S0196-0709(83)80068-4 6625103

[B65] Murphy C Gilmore MM Seery CS Salmon DP Lasker BR Olfactory thresholds are associated with degree of dementia in Alzheimer's disease Neurobiol Aging 1990 11 4 465 469 10.1016/0197-4580(90)90014-Q 2381506

[B66] Mattis S Bellak L, Katasu TB Mental status examination for organic mental syndrome in the elderly patient Geriatric psychiatry: A handbook for psychiatrists and primary care physicians 1976 New York: Grune and Statton 77 121

[B67] Sundermann EE Gilbert PE Murphy C Apolipoprotein E epsilon 4 genotype and gender: Effects on memory Am J Geriatr Psychiatry 2007 15 10 869 878 10.1097/JGP.0b013e318065415f 17911364

[B68] Zamora R Bartholow J Green E Morgan CD Murphy C Adiposity measures predict olfactory processing speed in older adult carriers of the apolipoprotein E4 allele Clin Neurophysiol 2011 10.1016/j.clinph.2011.09.001 PMC3691270 22055839

[B69] Murphy C Schubert CR Cruickshanks KJ Klein BE Klein R Nondahl DM Prevalence of olfactory impairment in older adults JAMA 2002 288 18 2307 2312 10.1001/jama.288.18.2307 12425708

[B70] Thesen T Murphy C Age-related changes in olfactory processing detected with olfactory event-related brain potentials using velopharyngeal closure and natural breathing Int J Psychophysiol 2001 40 2 119 127 10.1016/S0167-8760(00)00157-4 11165350

[B71] Kaplan E Goodglass H Weintrab S The Boston Naming Test 1983 Philadelphia: Lea and Febiger

[B72] Green J Levey AI Event-related potential changes in groups at increased risk for Alzheimer disease Arch Neurol 1999 56 11 1398 1403 10.1001/archneur.56.11.1398 10555661

[B73] Olichney JM Taylor JR Gatherwright J Salmon DP Bressler AJ Kutas M Iragui-Madoz VJ Patients with MCI and N400 or P600 abnormalities are at very high risk for conversion to dementia Neurology 2008 70 19 1763 1770 18077800 10.1212/01.wnl.0000281689.28759.ab PMC3071795

[B74] Chapman RM Nowlis GH McCrary JW Chapman JA Sandoval TC Guillily MD Gardner MN Reilly LA Brain event-related potentials: diagnosing early-stage Alzheimer's disease Neurobiol Aging 2007 28 2 194 201 10.1016/j.neurobiolaging.2005.12.008 16430992 PMC2631360

[B75] Han SD Houston WS Jak AJ Eyler LT Nagel BJ Fleisher AS Brown GG Cory-Bloom J Salmon D Thal LJ Bondi MW Verbal paired-associate learning by APOE genotype in non-demented older adults: fMRI evidence of a right hemispheric compensatory response Neurobiol Aging 2007 28 2 238 247 10.1016/j.neurobiolaging.2005.12.013 16434125 PMC1705815

[B76] Lind J Persson J Ingvar M Larsson A Cruts M Van Broeckhoven C Adolfsson R Backman L Nilsson LG Petersson KM Nyberg L Reduced functional brain activity response in cognitively intact apolipoprotein E epsilon 4 carriers Brain 2006 129 1240 1248 10.1093/brain/awl054 16537568

